# Weight changes during chemotherapy for breast cancer

**DOI:** 10.1590/S1516-31802002000400005

**Published:** 2002-07-07

**Authors:** Luciano José Megale Costa, Paulo César Spotti Varella, Auro del Giglio

**Keywords:** Breast Neoplasm, Chemotherapy adjuvant, Body weight, Obesity, Neoplasias, Mama, Quimioterapia Adjuvante, Peso, Corpóreo, Obesidade

## Abstract

**CONTEXT:**

Patients receiving adjuvant chemotherapy for breast cancer have a tendency to gain weight. This tendency has determining factors not completely defined and an unknown prognostic impact.

**OBJECTIVE:**

To evaluate weight change during chemotherapy for breast cancer in a defined population and to identify its predisposing factors and possible prognostic significance.

**DESIGN:**

Observational, retrospective cohort study.

**SETTING:**

Private clinical oncology service.

**PARTICIPANTS:**

106 consecutive patients with breast cancer treated between June 1994 and April 2000, who received neoadjuvant (n = 8), adjuvant (n = 74) or palliative (n = 24) chemotherapy.

**INTERVENTION:**

Review of medical records and gathering of clinical information, including patients' body weights before treatment and at follow-up reviews.

**MAIN MEASUREMENTS:**

Body weight change, expressed as percentage of body weight per month in treatment; role of clinical data in weight change; and influence of weight change in overall survival and disease-free survival.

**RESULTS:**

Therewas a mean increase of 0.50 ± 1.42% (p = 0.21) of body weight per month of treatment. We noted a negative correlation between metastatic disease and weight gain (r = -0.447, p < 0.0001). In the adjuvant and neoadjuvant therapy groups there was a mean weight gain of 0.91 ± 1.19 % (p < 0.00001) per month, whereas in the metastatic (palliative) group, we observed a mean loss of 0.52 ± 1.21% (p = 0.11) of body weight per month during the treatment. We did not observe any statistically significant correlation between weight changes and disease-free survival or overall survival.

**CONCLUSIONS:**

Women with breast cancer undergoing adjuvant or neoadjuvant chemotherapy gain weight, whereas metastatic cancer patients will probably lose weight during palliative chemotherapy. Further studies are needed in order to evaluate the prognostic significance of weight changes during chemotherapy.

## RESUMO

**CONTEXTO:**

Pacientes recebendo quimioterapia adjuvante por câncer de mama têm tendência a ganhar peso. Esta tendência tem fatores determinantes não completamente definidos e seu significado prognóstico é incerto.

**OBJETIVO:**

Avaliar a variação de peso durante tratamento quimioterápico por câncer de mama em uma população definida, identificar fatores predisponentes e seu possível significado prognóstico.

**TIPO DE ESTUDO:**

Observacional, de coorte retrospectiva.

**LOCAL:**

Serviço privado de oncologia clínica.

**PARTICIPANTES:**

Cento e seis pacientes consecutivas com câncer de mama tratadas entre junho de 1994 e abril de 2000 e que receberam quimioterapia neoadjuvante (n = 8), adjuvante (n = 74) ou paliativa (n = 24).

**PROCEDIMENTOS:**

Revisão de prontuários médicos com coleta de dados clínicos incluindo peso corporal das pacientes antes do tratamento e nas avaliações subseqüentes.

**VARIÁVEIS ESTUDADAS:**

Variação de peso corporal expresso em percentual do peso corporal por mês em tratamento, influência de variáveis clínicas nesta variação de peso e influência da variação de peso na sobrevida global e sobrevida livre de doença.

**RESULTADOS:**

Houve um ganho de peso médio de 0,50 ± 1,42% (p = 0,21) do peso corpóreo por mês de tratamento. Constatamos uma correlação negativa entre presença de doença metastática e ganho de peso (r = -0,447, p < 0,0001). No grupo em quimioterapia adjuvante e neoadjuvante, houve um ganho de peso médio de 0,91 ± 1,19 % (p < 0,00001) do peso corpóreo por mês de tratamento, enquanto que, no grupo com doença metastática (paliativo), observamos uma perda de peso média de 0,52 ± 1,21% (p = 0,11) do peso corpóreo por mês de tratamento. Não observamos correlação estatisticamente significante entre variação de peso e sobrevida livre de doença ou sobrevida global.

**CONCLUSÕES:**

Mulheres com câncer de mama recebendo tratamento quimioterápico adjuvante e neo-adjuvante ganham peso, enquanto mulheres com câncer de mama metastático possivelmente perdem peso durante quimioterapia paliativa. Estudos subseqüentes são necessários para avaliar o valor prognóstico da variação de peso durante quimioterapia.

## INTRODUCTION

Patients receiving adjuvant chemotherapy for breast cancer treatment have a tendency to progressively gain weight, as has been previously described in the literature.^1-8^ In these series, between 50% and 96% of patients had an increase in their weight, with 20% of them gaining more than 10 kg.^1^

Several factors may play a role in this setting, such as increased food intake due to anxiety or to alleviate chemotherapy-related nausea and emesis,^4,8,9,10^ decreased physical activity,^11,12,13^ and modification of basal metabolic rate.^1,14^ In addition, being premenopausal^1,4,7,8^ and the use of corticosteroids containing chemotherapy proto- cols^1,3^ have also been identified as risk factors for weight gain.

Regardless of the mechanism involved, weight gains may predispose these patients to chronic diseases such as arterial hypertension, diabetes mellitus and osteoarthritis, thereby negatively influencing their quality of life^6^ and possibly their survival. In addition, in some series, weight gain during adjuvant chemotherapy may correlate with tumor relapse.^1,15^

Breast cancer has a high incidence and is the leading cause of cancer death among women in Brazil (Datasus, 1999). Despite one previous paper that investigated the correlation between body weight and breast cancer incidence in Brazil,^16^ there is little data on weight changes observed during chemotherapy in breast cancer patients in our country. We therefore decided to make a retrospective study to characterize weight changes during chemotherapy among a group of consecutive women with breast cancer followed up by a single Brazilian institution. We also attempted to identify factors that would predict weight changes in these patients as well as investigating possible associations between weight changes and risk of tumor relapse and overall survival.

## Methods

We performed a retrospective observational review-based study of the medical records of consecutive women with breast cancer seen at a private clinical oncology service between June 1994 and April 2000. Only records containing complete information regarding tumor staging, menopausal status, initial anthropometrical data, therapy protocol, number of cycles (if chemotherapy was used), and at least two weight records (during one or more cycles of chemotherapy with at least one month between them), were considered. The patients were weighted during medical visits that were held during the afternoon hours, while using light clothes and always before the next chemotherapy session.

Following the aforementioned criteria, we selected for analysis 106 out of a total of 198 medical records. The major causes of exclusion were: no eligibility for chemotherapy (n = 24; 12.1%), drop-out (n = 39; 19.6%), and unsatisfactory weight data (n = 29; 14.6%). The mean follow-up was 4.9 months (minimum of one and maximum of 27 months) with a total of 520 patient-months. Seventy-four patients received adjuvant, 8 neoadjuvant and 24 palliative chemotherapy for metastatic disease. The median age was 49 years (minimum of 26 and maximum of 78 years) and 50 women (47.2%) were premenopausal. The initial body mass index (BMI) ranged between 16.9 and 41.8 kg/m^2^ (median of 25 kg/m^2^). The chemotherapy regimens employed in this study were CMF (Cyclophosphamide, Methotrexate, 5-Fluo-rouracil), FAC (5-Fluorouracil, Adriamycin, Cyclophosphamide), FEC and AC (Adriamycin, Cyclophosphamide)^17^ and forty-three patients (40.5%) received an anthracycline-based protocol (Table 1). None of the patients received corticosteroids as a part of their chemotherapy regimens, except for anti-nausea prophylaxis.

**Table 1:** Characteristics of patient groups

	Adjuvant n = 74	Neoadjuvant n = 8	Palliative n = 24	All patients n = 106
Age*	49.5 (43-56.5)	45.5 (39.7-53.5)	54 (41-62.7)	49.5 (43-59)
Premenopausal (%)	44.6	62.5	50	47.2
ER (%)	62	62.5	40	57
PR (%)	50	75	39	49.5
Axillary Ln+ (%)	52	87.5		
Weight*	61.8 (56-71.7)	57 (51.3-68.5)	65.8 (56.6-84.5)	61.5 (56-72.7)
BMI*	25.1 (22.3-28.6)	23.5 (21.8-27.5)	27 (22.6-34.1)	25 (22.3-29.2)

* *Express as median (interquartile range); ER = Expression of estrogen receptor in tumor; PR = Expression of progesterone receptor in tumor; Axillary Ln+ = Axillary lymph nodes involved by cancer; BMI = Body mass index.*

Univariate and multiple regression analysis were performed to investigate the role of age, clinical stage, menopausal status, use of an anthracycline-based protocol and initial BMI, as the independent variables, with weight changes as a dependent variable. We used Kaplan-Mayer curves and the log-rank test to evaluate whether there was a statistically significant effect from weight changes on the disease-free and overall survival. Values of p < 0.05 were considered significant.

## Results

Considering all the patients (n = 106), there was a mean weight gain of 0.50 ± 1.42% of body weight per month of therapy (p = 0.21), ranging from -4.51% to 4.43%/month. The presence of metastatic disease was a strong negative predictor of weight gain (r = -0.446, p < 0.0001). No other analyzed factor correlated significantly with weight gain when all the patients undergoing chemotherapy were considered together (Table 2).

**Table 2:** Factors predicting weight gain – all patients

Variable	Univariate analysis		Multivariate analysis	
	r^2^	^p^	r^2^	^p^
Age	-0.176	0.071	-0.219	0.098
Metastatic disease	-0.468	<0.0001	-0.446	<0.0001
Menopausal status	-0.004	0.968	0.080	0.541
Use of anthracycline	-0.080	0.413	-0.065	0.464
BMI	-0.062	0.536	0.002	0.985

*BMI = Body mass index.*

**Figure 1:**
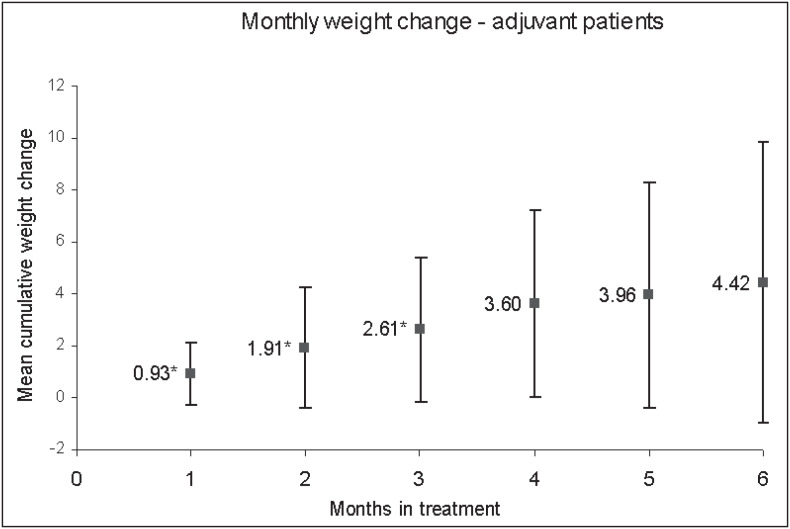
Mean monthly weight gain from initial body weight in patients receiving adjuvant chemotherapy

When the patients receiving adjuvant chemotherapy were considered as a group (n = 74), 60 patients (81.1%) presented weight gain while on chemotherapy and 10 women (13.5%) had a weight gain of over 2% per month. The mean monthly weight gain was 0.91 ± 1.19 % of initial body weight (p < 0.00001), ranging from -2.60% to 4.44%/ month (Figure 1). There was a tendency towards lower ages experiencing greater weight gain (r = -0.279, p = 0.069) (Table 3).

Kruskal-Wallis test performed; p < 0.000001; (*) Significant difference from other months.

**Table 3:** Factors predicting weight gain – adjuvant chemotherapy group

Variable	Univariate analysis		Multivariate analysis	
	r^2^	^p^	r^2^	^p^
Age	-0.228	0.051	-0.279	0.069
Menopausal status	-0.077	0.512	0.097	0.511
Use of anthracycline	0.100	0.396	0.033	0.805
BMI	-0.008	0.948	0.041	0.740

*BMI = Body mass index.*

**Figure 2:**
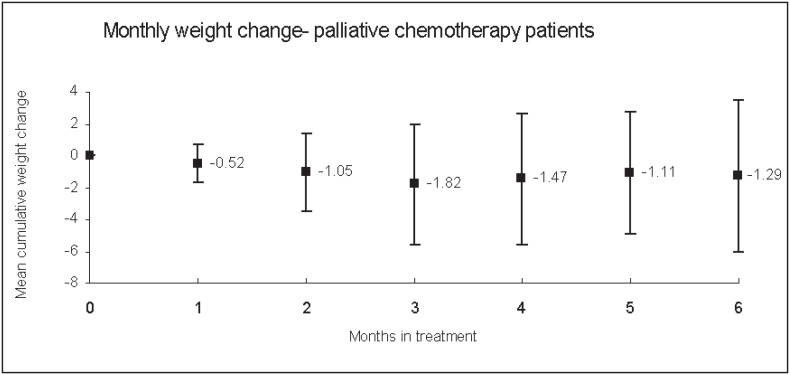
Mean monthly weight gain from initial body weight in patients receiving palliative chemotherapy for metastatic disease

In the group of women undergoing palliative chemotherapy for metastatic disease (n = 24), 12 patients (50%) lost weight while on chemotherapy. The mean monthly weight change was -0.52 ± 1.21% (p = 0.11) of initial body weight, ranging from 3.7% to 1.24%/month (Figure 2). None of the factors considered were predictive of weight changes in this group (Table 4).

*Kruskal-Wallis test performed; p = 0.11*.

**Table 4:** Factors predicting weight gain – palliative chemotherapy group

Variable	Univariate analysis		Multivariate analysis	
	r^2^	^p^	r^2^	^p^
Age	-0.130	0.544	-0.192	0.714
Menopausal status	-0.090	0.677	0.271	0.618
Use of anthracycline	-0.223	0.294	-0.261	0.322
BMI	-0.099	0.644	-0.162	0.557

*BMI = Body mass index.*

**Figure 3:**
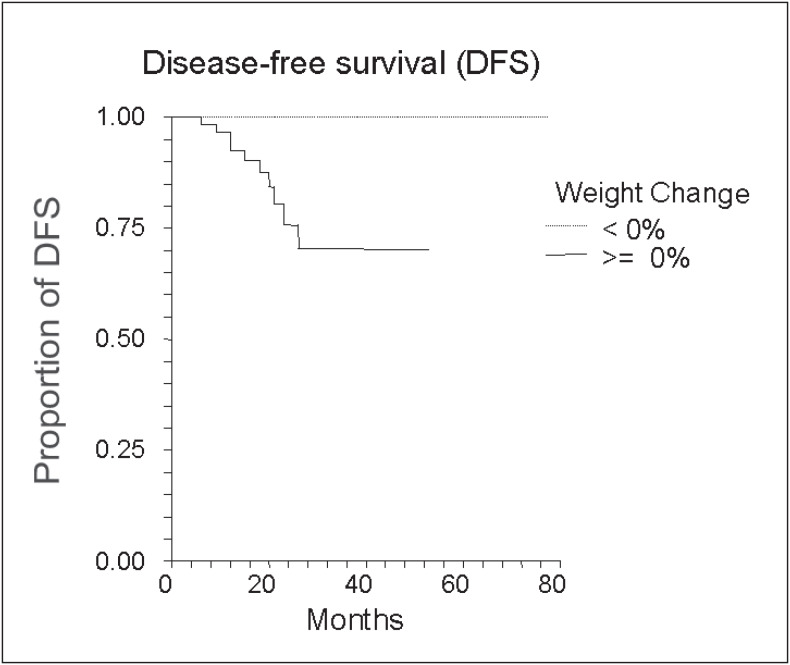
Weight gain and disease-free survival in patients in adjuvant chemotherapy

**Figure 4:**
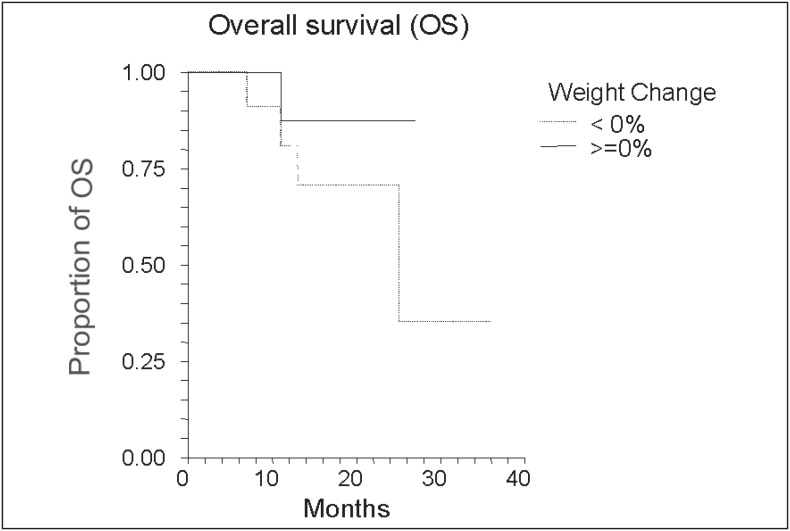
Weight loss and overall survival in patients in palliative chemotherapy

We found no statistically significant correlation between weight gain and disease-free survival in the group of women receiving adjuvant chemotherapy (Figure 3) or with weight loss and overall survival in the metastatic breast cancer group (Figure 4).

*p = 0.08; log-rank test performed*.

*p = 0.12; log-rank test performed*.

## Discussion

Several authors have already reported the occurrence of weight gains during adjuvant chemotherapy for breast cancer.^1-7^ Our similar findings in a series of patients treated at a Brazilian institution strengthen this association, with the lessening of the role of potential regional dietary or environmental factors.

The weight gains that we observed in this study are thought to be of sufficient magnitude to foster a negative impact on corporal self-image, self-esteem and, consequently, the quality of life of the patients studied.^6^ Therefore, the attending physicians who care for such women need to be aware of this fact and may need to institute preventive measures such as exercise programs and dietary interventions to decrease weight gains during adjuvant chemotherapy.^13^

In our study no statistically significant correlation was found between weight gain and shorter disease-free or overall survival. Likewise, in the literature it was difficult to find convincing evidence for an association between weight gain during adjuvant chemotherapy and risk of relapse. In spite of positive conclusions from some series,^1,18,19^ this possible "new" risk factor for relapse has not been confirmed in other papers^3,4,20^ and this issue is therefore not settled definitively. The supposed biological plausibility for this phenomenon among obese women rests on greater peripheral conversion of androstenedione to estradiol and inhibition of synthesis of sexual hormones binding globulin with a consequent increase in levels of free estradiol which stimulates residual neoplastic cells.^21-24^

Among patients undergoing palliative chemotherapy we found a tendency towards weight loss. In these women weight loss is probably associated with cachexia, anorexia or symptoms determined by the presence of advanced disease,^25^ with a possible negative impact on survival.

Further studies with a greater number of patients undergoing adjuvant treatment protocols and with a longer follow-up are necessary so as to better define the real prognostic impact of weight gain in this setting. This association, if confirmed, will be an important argument in favor of employing aggressive strategies to control weight during chemotherapy.

## Conclusion

Weight gain associated with adjuvant chemotherapy for breast cancer also occurs in our setting. Such an association was not found with palliative chemotherapy. The prognostic impact of weight changes associated with breast cancer chemotherapy remains unknown.
